# Mr. Howship on Diseases of the Urinary Organs

**Published:** 1823-09-01

**Authors:** 


*A Practical Treatise on the Symptoms, Causes, Discrimination, and Treatment of some of the most important Complaints that affect the Secretion and Excretion of the Urine. The first head, including Suppression; from Congestion, Inflammation, Calculi, Abscess, or other Diseases in the Kidneys; the Appearance of Blood, Pus, Albuminous Matter, or Gravel, in the Urine; and the various Kinds and Seats of Urinary Calculi. The second, specifying the Circumstances inducing Retention; in the Kidneys, Ureters, Bladder or Urethra; as Old Age, Paralysis, Gouty Spasm, Strangulated Rupture, Tumors in the Bladder, Hernia Vesicæ, Displacement or Pressure of other Viscera, Ruptured Bladder, Inflammation of Urethra, Gonorrhœa, Contusion, Tumors, Enlarged Prostate, Spasmodic, and Permanent Stricture: with Remarks on Puncture of the Bladder. The whole exhibiting a Comprehensive View of the various Diseases of the Kidneys, Bladder, Prostate Gland, and Urethra. Illustrated by numerous Cases, and Engravings.* By John Howship, Member of the Royal College of Surgeons in London, &c. &c. 8vo. p. 448, four plates, London, April, 1823.*

It is the object of Mr. Howship, in this work, to bring into as clear and comprehensive a form as possible, the most interesting results of his own experience, occasionally aided by that of others, on the particular subject in question. The disorders, he observes, that regard the secretion and excretion of the urine, form an extensive line, or rather circle of pathological research that has been too much neglected, partly from the feelings of false delicacy that have prevented these maladies from being complained of in their commencement —and partly from the neglect of sufficient post mortem examinations on the part of the practitioner. John Hunter, Sir Everard Home, Desault, and some other writers have done much towards the elucidation of the class of diseases in question; and of their writings Mr. Howship occasionally avails himself; but "the observations adduced, and the principal rules grounded upon them, have been derived al-

* We make it a general rule to give the whole of the title page of a work when it is reviewed; but Mr. Howship must see how very aukward a long title page looks, either in a book or in a review. We advise authors to avoid the practice of making the title page a table of contents.

most exclusively from his own experience in these diseases." For many of the pathological illustrations he is indebted to his friend and patron Mr. Heaviside, to Dr. Hooper, to the College Museum, and to the Collection in the Military Hospital at Chatham. To the Museum of Mr. Brookes our author also acknowledges himself indebted, especially in the pathology of the kidney.

Such being the substance of the introductory observations we shall now proceed to the practical matter itself.

PART I.—*On the Disseases that Affect the Secretion of Urine.*

THE first chapter is on *suppression* of urine, the symptoms and diagnostic marks of which are laid down by our author with perspicuity and conciseness. The causes of this dangerous malady are considered to be, in general, local congestion, inflammation, calculi, and abscess in the kidney. He believes with Desault, that Nature endeavours, in such cases, to procrastinate the fatal event by throwing a vicarious urinous discharge from other emunctories, as the skin, stomach, bowels, &c.

"Mention is made by Dr. JOHNSTONE, of a very curious fact, in a case of suppression, in which, 'for some days before death, the skin was all over as white as if it had been powdered. This white dust being gathered, it was found to have the taste of crude sal ammoniac.' The mode in which this was explained, was 'the secretion of urine prevented, the perspirable matter became so supersaturated with ammoniacal salt, that it crystallized upon the skin.' But the most singular instance upon record, perhaps, is related by Dr. DAWSON, of a woman, in St. George's Hospital. Her complaints were various and severe, and with other symptoms, after several temporary attacks, came on a permanent suspension of all action in the kidneys, and she is stated to have had a total suppression of urine for above fifteen months. During this period, she frequently vomited every day, sometimes every two or three days. If the vomiting came on after eating, what was rejected seemed to be mere urine, without any mixture of what had been taken. By occasional purges and other means, œdematous swellings of the limbs were kept under. Her breasts became ailing, and discharged a watery fluid, which, like the other discharges, had a urinous smell. At length uncommon pricking pains were felt all down the back and loins, and about the belly and groin, with great heat. On the second day she voided three ounces of thick slimy matter, with sharp pains in the urinary passages; this water was not high coloured. The next day she passed healthy urine. Afterward she often had a suppression of urine ten or fourteen days, and once for two months, during which she had no vomitings; but her body was very much swelled."* 4.

* Philosophical Transactions.

It must be difficult, we imagine, to determine when suppression of urine owns for its cause *congestion* of the venal vessels, unless where blows or other local injuries have preceded the complaint. In such cases the treatment will be obvious enough.

The causes of suppression from inflammation will be, violent impressions of cold—acrid, irritating medicines, as cantharides, turpentine, &c.—the irritation of calculi, or of gout in the system. This last our author considers to be a very frequent cause of the complaint under consideration.

"A case of complete suppression from gout, terminated happily under Mr. Heaviside's care; and was the only instance of recovery from complete suppression, he had ever seen. A general officer, walking home on a cold night, from the House of Commons, in full dress, with gout in his foot, the complaint left his toe, and the next day, Thursday, with great pain in the loins, he made little water, on Friday less, and on Saturday none. On *Sunday*, with Sir Francis Milman, Mr. Heaviside visited him: he felt the abdomen, but found no tumour; requested to draw off the urine; he passed a catheter, and found, as he expected, the bladder empty. Terebinthinate medicines were directed, and, on taking the third dose, the patient felt desire, and passed nearly a pint of water. Sent afterward down to drink the waters at Bath; he there had a regular fit of the gout, and perfectly recovered." 9.

The appearances on dissection, if notable, will most frequently present some stage or consequence of suppuration, and will rarely be confined to mere vascularity. The treatment, in this kind of suppression, will be the antiphlogistic, of course, as bleeding, the warm bath, mild purgatives, emollient glysters, and demulcent liquids. Blisters, so generally objected to in nephritis, our author has never found injurious, and almost invariably advantageous.

At page 13, our author relates a case where gout made its attack on the kidney, in the marked form of partial suppression of urine; but shifting to the toe the renal symptoms immediately subsided.

The third section treats of suppression from calculi in the kidnies. The mode in which calculi act in causing suppression of urine is not well ascertained—probably from irritation. In general, there is either high vascularity of the membrane lining the pelvis of the organ—ulceration of its internal surface—or some other organic injury, when the suppression takes place.

"A woman labouring under these complaints, died with violent pain in the left side. For five or six weeks she had complained of pain in making water, and constantly discharged, with little urine, a large quantity of matter. Her complaints altogether had lasted seven or eight months. A great quantity of purulent matter was found among the intestines, in the left side of the abdomen; none in the right. The left kidney was almost entirely destroyed by long-continued irritation, and consequent ulceration. Only a part of the pelvis remained, which was thickened, scirrhous, and contained two stones; one thick, and with processes extending into the infundibula; the other, a small calculus. The lower ribs, on which the kidney had laid, were carious. The bladder was healthy, but full of pus." 15.

It is well known that, at a certain period of suppression of urine, the renal affection excites disturbance of the stomach, and this disturbance is usually followed by affection of the head, of a comatose character, and attended with serous effusion within the cranium.

In respect to the treatment of suppression from calculi, the depletory at first, and demulcent or anodyne afterwards, will be the most rational. Where little or no pain is felt about the loins, it might be prudent to try the more powerful terebinthinate remedies carefully watching their effects.

"In the enumeration of symptoms common to suppression, (2,) it was observed to be usually unattended with desire to void urine, and unconnected with tumour in the seat of the bladder. Professor Delpech very truly observes, that there are cases in which, from one symptom being absent, and another indistinct and obscure, the treatment will require particular care. Thus, retention of urine may be connected with rigid spasm of the abdominal muscles, so as to conceal the tumor of the bladder; and, on the other hand, hysterical affections, with suspended secretion of urine, and at the same time the most incessant and urgent calls to pass water, may be also attended with similar spasm of the muscles of the lower belly; and this continuing several days, with great pain, and no flow of urine, may lead the attendants to conclude it is retained in the bladder, while the tense abdomen prevents the detection of the tumor." 17.

Mr. Howship has known considerable relief obtained by keeping up a drain, by issue or blister, from the loins. Some cases in illustration are adduced by our author, and to these we must refer the reader for particulars.

Mr. Howship devotes a pretty long section to abscess of the kidney and illustrative cases, which well deserves the practitioner's perusal. It is, however, not adapted for analysis. The following case is so extraordinary, and involves so interesting a problem in physiology, that we shall give it entire.

"A young lady, aged 24, first observed an enlargement in the abdomen, in 1816, slowly increasing, till it equalled that of an eight months' pregnancy; which tumor, with occasional variation, remained. *November* 5, 1818, she first had retention of urine, for which, a catheter was passed, and two quarts of water drawn off. The instrument was regularly introduced, but the quantity of urine decreased, so that on *November* the 10th, only a dessert-spoonful passed. This state of things continued till the 8th of *December*, while powerful medicines were given, the warm-bath tried twice, and electricity four times, with little benefit, as there never was more than an ounce or an ounce and a half of urine drawn off every two or three days. But on *December* the 8th, a sudden influx took place, and lasted 20 days, during which 17 gallons, and half a pint of water passed from the bladder by the catheter. *December* the 29th, the urine again ceased to flow into the bladder, and so continued till *March* the 2d, 1819, except on two days, (the 1st and 2d of *February*, (on which days, one pint a day was drawn off. From *March* the 2d to *April* the 6th, a tolerably natural quantity of water was voided by the catheter. From *April* the 6th to *May* the 30th, no water flowed into the bladder. The catheter passed once in two or three days, scarcely a tea-spoonful was ever found. Towards the close of this period, however, a new change took place, for, *May* the 25th, a discharge of water came on, *by the bowels*; and between the 25th and 29th, 16 pints of water were passed. This ceased the day before the next influx into the bladder, which, as before stated, was on *May* the 30th. The discharge of water from the bladder now continued till *June* the 10th. During this period, ten and a half gallons of water passed, most of which was forced from the bladder by violent spasms, The cessation of flow into the bladder had returned only two days, when the urine again found its way *by the rectum*, and so continued till *August* the 12th, during which time seven and a half gallons were voided. An influx now again took place into the bladder, and continuing six days, near eight gallons were drawn off by the urethra, and one and a half gallons passed by the *bowels*. After the water ceased to flow into the bladder, ten and a half pints again passed by the bowels, five and a half on the 19th, and five on the 20th of *August*, when the retention returned, and no urine passed either way, till on *September* the 8th, she wrote to Mr. Heaviside 'That she could hot help fearing, from her present sensations, that an influx was again about to take place in the course of a day or two.'—*September* 14. Within the last three days, the influx having taken place as she predicted, twenty-two quarts were passed from the bladder in occasional spasmodic gushes.—*December* 6. For four days in the last week, she passed *two gallons of urine a-day by the rectum*, while for the preceding fortnight, not two table-spoonfuls in the whole either passed by the bowels, or reached the bladder.

"*May* 21, 1821. Mr. Heaviside said she remained just the same; sometimes not voiding a quarter of a pint in a fortnight, and then either by the rectum or bladder, three or four gallons in a day.

"*August* 17, 1822. Mr. Heaviside acquainted me the above complaints remained much the same, the general health being perfectly good. Sometimes two gallons of urine a-day were voided by the bowels; and sometimes when the influx had taken place, unless regurgitation occurred, the urine was obliged to be drawn off, four or eight ounces in the day; but if the influx happened in the afternoon, and her surgeon was prevented calling the same evening, by the next morning regurgitation had taken place, and not a drop was found in the bladder. This had happened very frequently. 50.

As there can be no idea entertained of a direct communication, in this case, between the bladder and rectum—and if Mr. Howship's and Mr. Heaviside's statements be correct, that *urine* was passed by the *rectum* on several occasions, it must follow that a regular secretion, for instance, urine, may be taken up from its proper receptacle, and discharged unaltered by some other outlet—or, that a *vicarious* secretion, *similar to the natural one*, may take place in the system. Considering the litigations which this question has undergone, and the acrid controversies to which it has given rise, we think Mr. Howship was bound to ascertain the fact stated above in a manner that might have settled the dispute. We hope he examined and analyzed the *urine* that came per anum—for if it was really urine, it might have been equally as well deposited in the stomach, or any other part of the body.

Chap. II. *Morbid Appearances in the Urine.* The first section of this chapter is on bloody urine. Although where this proceeds from the kidney, there is generally some injury of its internal cavity, yet it does not necessarily follow that there should be rupture of vessels. The exhalents, and the secreting vessels of the kidneys have been known to pour out pure blood. The occurrence, however, is comparatively rare. The phenomenon usually depends on some disease of the urinary apparatus or foreign body lodged in some of the passages. The cause, therefore, must be sought out, and then the proper remedy will be evident enough. Several interesting cases are related by our author in this section.

The second section is on the appearance of *pus*, in the urine. It is not easy here, or indeed any where else, to distinguish pus from mucus. It is observed by Desault, that purulent matter does not, in every case, arise from disease in the urinary organs themselves; for that numberless obervations shew how readily acute diseases establish their crisis by the medium of this appearance in the urine. The same author adds, that a great number of facts attest that matter deposited in the lungs, liver, or any other part of the body, is capable of being carried by metastasis to the kidneys, and evacuated with the urine. As for Mr. Howship himself, although he has often seen these critical deposits in the urine; they have always consisted of some variety of albuminous matter, generally of a farinacious appearance, or of a pink sediment, or some modification of gravel. Nor has he seen an unequivocal instance of the contents of an abscess being transferred from other parts by metastasis to the kidneys.

"The doubts just expressed in relation to critical deposits by the urine, apply equally to many of the appearances that originate in irritation of the membrane lining the cavities either of the kidneys, ureters, bladder, or urethra. Where, from inflammation, abscess of these parts takes place, and from breaking inwardly, the contents are evacuated with the urine, the matter will of course be, strictly speaking, purulent. In gonorrhœa, also, I am aware the discharge, without breach of surface, is purulent, because I have seen, as well as others, that under the microscope, it is impossible to distinguish this matter from that of an abscess, the contained globules, as to number, colour, and magnitude, being precisely the same in both cases, that is, nearly similar to those found in the blood. But in most of those complaints in which the urine is clouded, in consequence of some irritation in the kidneys, the substance deposited will be frequently very apt to be mistaken for pus, while, in fact, it only resembles it in colour. Examining some such urine very lately, I found the sediment extremely tenacious, of a deep yellow colour, and even consistence, circumscribed, partly suspended in the urine, like a little undulating cloud, and partly attached to the bottom of the vessel. It appeared to me a muco-purulent matter; but on the affusion of a quantity of hot water, the whole became slightly turbid: in a few minutes the fluid cleared, was poured off, and on examining the sediment, which answered to the quantity of the previously existing cloud, it proved to be a fine, dense, beautifully white, flocculent, albuminous substance, before disguised, but now destitute of any trace of resemblance to purulent matter." 66.

The third section of this chapter is on the appearance of *albuminous* matter in the urine. This is a very frequent occurrence, and is sometimes not to be detected but by chemical analysis. It is, however, only when it is apparent to the eye that our author applies the following remarks:—

"The most frequent and least important cause of this appearance is the occasional effect of common cold, in which, after a sense of weight and irksome uneasiness about the loins, the urine is voided turbid, but on standing deposits a greater or less quantity of fine whitish-coloured flocculent matter, not very much exceeding in specific gravity the fluid in which it slowly subsides; generally disappearing in the course of a few days.

"Analogous to this appearance, is that which is observable upon the crisis of feverish indisposition. In these affections the sediment is generally tinged more or less decidedly either of a pink, or a dull red colour; the principal seat of irritation, in all these cases, I be-believe to be the mucous membrane lining the cavities of the kidneys.

"An attack of gout is commonly productive of some disturbance to the circulation through the kidneys, being attended with a flocculent pink coloured albuminous deposit in the urine. It also occasionally happens, that from some accidental circumstance, the gouty action is in a more direct manner transferred to the kidneys, and the consequence is more considerable disturbance, or a complete suspension of their functions." 70.

Deposits of the kind in question are occasionally observed in the urine from temporary irritation at the neck of the bladder. A transparent and extremely viscid albuminous substance, also, occurs in urine, yet not derived from affection of the bladder, but from enlargement of the prostate gland.

"According to my experience, in the former case, this appearance of transparent matter has been only occasional and transitory, furnished in small quantity, not perfectly pellucid, of low specific gravity, and not very tenacious; in the latter it is, on the contrary, a permanent appearance, furnished in much larger quantity, beautifully transparent, of high specific gravity, and so tenacious as to admit of being raised in threads several feet in length. M. Desault considers that 'Les urines glaireuses sont un symptôme propre aux affections de la vassie.' A conclusion which I believe to be at variance with the general results of English practice; it may, however, perhaps, be explained by taking into account the difference of climate and modes of life in France and England; circumstances calculated to qualify the state of constitution, and influence the appearances of disease, as well as the functions of health." 74.

The treatment of these albuminous deposits in the urine will vary with the cause. It will generally be found that the membrane lining the internal cavities of the kidney is suffering some irritation, independent of ulceration or calculous tendency.

"Under these circumstances, I have occasionally found various medicines useful, although it is extremely difficult to lay down any general rule for their application. In one instance opiates and other anodynes were sometimes conducive to comfort, but the most powerful and permanent relief was derived from the recently expressed juice of the lemon. (104.) In other instances, no medicines have appeared to me capable of making any decided impression. Tonics, in general, I am inclined to believe, are among the most useful means. Preparations of steel, particularly the muriated tincture, have obtained, in these complaints, a very high character with some excellent practitioners, particularly with Dr. Hooper; and I have certainly sometimes, though not often, directed them with advantage." 75.

Some good cases are appended to this section by Mr. Howship.

The 4th section is on the *appearance of gravel in the urine.* The morbid affections of the urine above described may occur either as the consequence of accident, or the effect of disease; but other depositions are observed, that shew some derangement in the functions of the secreting vessels of the kidneys—" probably connected with imperfect assimilation of some of the principles of the blood." Our author considers the profession greatly indebted to the researches of Wollaston, Brande, Marcet, and Prout, though it is to be confessed that many points in the history and treatment of calculous disorders yet stand in need of elucidation. We must pass over a great portion of Mr. Howship's work on calculous affections, as he does not profess to exhibit any particular discovery or new information on this subject—though he brings forward much valuable matter for the perusal of the junior branches of the profession.

At page 155 of this volume, Mr. Howship enters on the consideration of " irritable bladder"—a troublesome and important disease. The symptoms are well known to be more or less of uneasiness in the organ, attended with increased frequency in making water, and a discharge of mucous or albuminous matter from the membrane lining the bladder. Sometimes the above symptoms are attended with urgent tenesmus and straining, so much aggravated in the expulsion of the last drops of water, that the turgid vessels on the membrane lining the neck of the bladder occasionally give way, and hæmorrhage follows. Mr. Howship here enters into a consideration of *wounds* of the bladder, quoting freely from Larrey and other surgical writers. This is against all arrangement, and embarrasses us exceedingly when attempting to follow up the leading subject of the section. Wounds and other injuries will doubtless prove causes of irritable bladder, which is not a disease then, but a symptom; and it is not necessary to go into the minute history of those primary affections of which irritable bladder is only a sequence.

Our author considers cold as only rarely a cause of irritable bladder. " A much more common, and indeed one of the most frequent causes of irritation in the bladder is a disposition to deposit gravel in the urine." Affections of the neighbouring parts, as of the prostate gland, uterus, vagina, or rectum, will occasionally produce the complaint in question.

" The coats of the bladder sometimes become subject to spontaneous disease. When the inner membrane alone is thus affected, it commonly exhibits either effusion, ulceration, or circumscribed tumors, generally prone to assume, sooner or later, the characters of fungous disease. An instance of this, connected with extreme irritation from gravel, will be presently related; and Mr. Watson mentions opening the body of a middle-aged man, whom he sounded in his lifetime for stone, but could find none. He had, however, the symptoms of stone, and frequently passed bloody water, especially for many days before death; which took place from inflammation in the bowels. On examination, the left kidney was small, extenuated, and internally formed into lobuli, but containing neither stone nor gravel. The urinary bladder was flaccid, and almost empty. On cutting it open, 'it was full of fungous tubercles.' The prostate gland was greatly enlarged and indurated." 165.

Le Cat relates a terrible case of this disease in a female, who had long suffered from pains in the back and loins, with pain and irritation in the bladder. These symptoms were referred to calculus, but the sound gave no satisfactory information. The surgeon, however, at last cut into the bladder with the hope of finding a stone, or, at least, of removing fungous excrescences. No stone was found, but several clusters of excrescences were felt, one of which was of firm texture. Many of these were extracted with the forceps. The patient suffered extreme pain, and violent fever followed, of which she died.

Mr. Howship does not lay down any distinct principles of treatment, concluding, we suppose, that the cases in illustration will be sufficient. This, however, precludes us from giving a connected review of the work. We shall here introduce a single case, because it is a good specimen of those troublesome affections about the urinary and genital organs which we often meet with in young persons, whose generative apparatus is under a morbidly irritable condition.

" *April* 20, 1821. I was consulted by a young gentleman, who had lately graduated in the University of Edinburgh. His complaints were a perpetual source of distress. From early youth, of irritable habit, he had been subject to frequent nocturnal emissions, occasional sharp pain in the glans penis, and acrimony of urine; these circumstances he sometimes attributed to close study, at other times to bad digestion. Frequently he felt dull aching pain in the perineum, after some months extending itself by degrees to the bladder. He thought the bladder easiest when empty, but scarcely uneasy when it contained a pint of urine; he never had occasion to pass it, during the night. Female society produced a painful sense of fulness and distention in the parts. The urethra and bladder had been repeatedly examined by elastic bougies, and by the sound. These examinations had always aggravated his sufferings. In conclusion, he had been much in the habit of taking opium, as it had afforded him a certain degree of relief. I requested him gradually to diminish his usual dose of opium; and directed some mild aperient medicines.

" *April* 28. Extreme uneasiness, and a most distressing sense of fullness in the perineum, had induced him to apply leeches, affording partial and transitory relief. He had been recommended warm baths, but as they uniformly made him worse, he laid them aside.

"In the beginning of *May* he left town for the West of England; where, at my request, he bathed several times in the sea, and took the muriated tincture of iron. He wrote to me on the 15th, stating what he had done, and adding that his distresses were rather increased than diminished; that he found himself much weakened by the harassing nature of his complaints. After bathing he experienced the glow of reaction, but felt nevertheless an absolute increase of pain in the bladder, and discomfort during the day. After expelling urine, he still felt the pungent stinging pain, almost sposmodic, in the glans; with increased aching in the perineum. The urine had long contained small filamentous appearances, and now exhibited these appearances more evidently. The functions of the stomach were badly performed, and the steel not agreeing well, he had laid it aside, substituting the bark. He particularly entreated to know, if I thought his complaints could depend on calculus. In my reply, I assured him I had no idea of any disposition to stone; but that I believed his complaints arose from a partial congestion of the vessels upon the inner membrane of the neck of the bladder, and prostate gland, in connexion with an irritable state of the seminal ducts, opening into the prostratal part of the urethra, and nothing more; and that I would recommend him to lay aside sea bathing, but to continue taking the bark.

"*Oct.* 31. He wrote, expressing much regret at not having continued the means directed, he said he had tried fifty plans, and adhered to none; adding that he had been punished for his unsteadiness. The urethra was now so extremely irritable, he felt sorry I had not thought it right to pass a bougie for him when in London; and operation which now he said was scarcely practicable. A diminished stream of urine, a diffused pain in the bladder, the old pain at the glans, frequent emissions, general debility, and impaired digestion, formed his present list of complaints.

"The concluding part of his letter, however, was the most important, in which he said, 'caustic has been proposed, and has been once applied, near the bulb, the other two strictures being passed more readily; but as yet without mitigating the symptoms. I am in doubt whether to have it employed again, or any other application.' To this I replied, that I should unquestionably advise him to come up to town, before any thing more was done.

"*Nov.* 14. He reached London, and to satisfy his mind, I at once passed a fair-sized bougie with perfect ease into the bladder. I carefully examined the bladder from the rectum, by the finger, ascertaining the condition of the more remote parts of the bowel by other instruments; without discovering disease, contraction, or irritation. The prostate gland and bladder felt very healthy; and the intestine, for eleven and a half inches of its extent, was perfectly sound. He could scarcely, however, believe the urethra free from stricture, and was therefore particularly desired not to leave town without another opinion, to which he assented. I therefore met the late Mr. Wilson in consultation, who passed a full-sized silver sound at once into the bladder. It satisfied him, and he now declared with amazement, that more than twenty surgeons, at Bath, Bristol, Clifton, and elsewhere, had passed bougies for him, without one being able to get beyond the bulb: while most of them agreed there were three strictures.

"The derangement in the association of the natural functions of the generative organs, was regarded by Mr. Wilson, as it had previously been by myself, as the true cause of all his complaints. He consequently gave him the same opinion he had before received, which was to avoid risk, and obtain comfort and security, by determining to marry early; regarding such change in circumstances, as a measure most likely to bring the functions and feelings of the system progressively round to their natural and healthy state; assisted occasionally by tonic medicines." 184.

We come now to Part II. of the work before us, the first chapter of which is on incontinence of urine—a complaint occurring principally in youth, although not unknown in later periods of life. The involuntary discharge of urine, during sleep, has been variously accounted for—our author thinks that—"a moment's consideration will clearly explain it." We shall allow Mr. Howship to give his explanation in his own words.

"The general muscular coat of the cavity of the bladder may be regarded as an involuntary muscle; while, on the contrary, the circular band of muscular fibres surrounding its neck is, to a certain degree, obedient to volition. Now we know very well, that a state of repose relaxes very much the whole system of voluntary muscles, exerting little or no influence over the system of involuntary movements. Upon this principle, it is evident an involuntary flow of urine might more readily occur during sleep, than while awake. There is, however, I believe, another circumstance tending to explain how it happens. In the majority of cases, the early age of the child prevents the ascertaining whether the urine flows during a state of positive oblivion, or whether this event takes place only under some particular mental impression. The latter state, I rather believe, is mostly an invariable condition. Intimately acquainted with a young person, in early youth long subject to this habit, he mentioned to me one circumstance that, upon these occasions, had often struck him as curious; it was, that he never at any time wetted the bed, unless when engaged in a dream he felt the accustomed uneasiness from desire to make water, and fancy, immediately supplying what was wanting in time and place, the act of voiding it became, in point of fact, as perfectly voluntary as at any other time." 203.

Mr. Howship observes that incontinence of urine in young persons is, in general, very easily removed. "All that is commonly necessary is to stimulate, to a certain degree, the neck of the bladder; and this is most conveniently accomplished by the application of a small blister to the loins, or, if that fail, to the perineum, the blister being for some time kept open, and dressed occasionally with the unguentum lyttæ." The same treatment is recommended where the complaint occurs in the adult subject, induced by fatigue of parts, from excessive debauchery, or perhaps the consequence of a slight paralytic affection. The cases we must pass over, referring the reader to the work itself for the particulars.

The second chapter is on retention of urine. Of retention in the ureter and kidney a number of examples are cited from different authors, in addition to our author's own range of experience. On retention in the bladder, we meet with many judicious observations; but they are more adapted to the junior than the senior branches of the profession, as containing plain elementary, not novel matter. It is on this account that we are obliged to pass over the greater part of the work entirely unnoticed in our review.

*Gonorrhœa.* Mr. Howship observes that it would be very easy to enlarge upon the subject of gonorrhœa; but that it would be unnecessary, "as the whole that is at present known, of practical importance, may be said in very few words." "It is clearly established," say he, "that the same matter which in one person produces gonorrhœa, will, in another, produce chancre; and that this difference depends on the disposition of the habit to favour the production of the one in preference to the other effect, of the venereal poison." We were not aware that this point was "clearly established"—on the contrary, we believe that the predominant feeling is against this identity of poison. If they were the same, we should in almost every case have the two forms, as all the parts are equally exposed; whereas we very rarely have the two forms in one individual.*

"As to the nature of the matter discharged in gonorrhœa, I have repeatedly examined it under the microscope, as well as most of the other varieties of purulent and albuminous excretions from mucous and ulcerated surfaces, without having been able to perceive any essential difference between pus taken out of an ulcer, and pus excreted from the surface of the inflamed urethra in gonorrhœa." 260.

---

* We quote the following passage from the latest systematic work in our own language. "We are chiefly indebted to Mr. Hunter for having pointed out the distinction, (between the poisons of syphilis and gonorrhœa,) and there is now scarcely an individual who has any doubt upon the subject, though there are several who conjecture that it has been derived from the syphilitic venom changed and softened in its virulence by an introduction into different constitutions. These conjectures are harmless, but they have little ground for support." *Good's Study of Medicine, Vol. IV. page 78.*

We shall give our author's practical indications in gonorrhœa, which are, upon the whole, judicious.

"Upon the first accession of the symptoms, the patient should place his diet under some restriction, dependant on state of constitution. If plethoric, he should lay aside or take but little wine or malt liquor, using little exercise, with an aperient draught night and morning. If the habit of body is less disposed to excitement, the restrictions may be less rigidly observed; but the bowels still kept open, with care to avoid fatigue.

"As the complaint advances, the symptoms increase, and chordee becomes generally one of the most incessant sources of distress. In this stage leeches may, in some cases, be applied with advantage, aided by light, saline, diaphoretic, and aperient medicines, directed at short intervals. The taking only mucilaginous decoctions, avoiding animal food, and keeping quiet, will generally procure much relief. Should these means not succeed, an anodyne draught will frequently operate well, in allaying irritation, and procuring quiet repose.

"Some practitioners are in the habit, from the first, of directing the liquor potassæ, continuing its use till the decline of the inflammatory symptoms. This medicine, however, is extremely uncertain in its effects. I have, in some cases, found it answer very well, but in others, it has excited uneasiness and irritation about the neck of the bladder, and more than once, much difficulty as well as straining, in voiding the urine. So that where I now direct this medicine, it is always in combination with some aperient, to prevent its remaining long in the bowels.

"Where the symptoms have reached their highest point, and are upon the balance towards decline, they will, for a short time, require more close attention. If the least constitutional disturbance, fulness of pulse, heat of skin, or thirst occur, the diet should be rendered still more abstinent, and the diluent system be brought still further into adoption.

"Should there, at this period, be any manifestation of increased uneasiness or tumor about the deeper seated parts of the urethra, no time must be lost, in preventing the continuance of such tendency; by confinement to bed, with saline diaphoretics in the strong, and compound powder of ipecacuanha in the weak; and also by the free use of various farinaceous decoctions. Where the continuance of these means scarcely arrest are progress of the threatened evil, leeches upon the parts, or cupping glasses near them, must be directed; followed by fomentations." 270.

When, in the course of a few weeks, and by the treatment just described, the inflammatory symptoms subside, and yet a discharge continues, our author ventures cautiously on terebinthinate or balsamic remedies, and has found himself generally successful.

The remainder of the volume is on retention of urine from various causes, as spasmodic and permanent stricture, &c.

and as the cases form the principal feature, we find it impossible to give a connected analysis. The matter is very valuable to the practitioner and to the student, and to these we recommend the publication, not as containing much novelty, but plain and useful directions, solid and authenticated facts. It is hardly necessary to say that the plates are admirably executed.

---

